# Single‐strand superficial medial collateral ligament reconstruction with a peroneus longus split graft provides reliable control of anteromedial rotatory instability combined with anterior cruciate ligament reconstruction: A 2‐year clinical cohort study

**DOI:** 10.1002/jeo2.70941

**Published:** 2026-10-05

**Authors:** Philipp Weber, Simone Maxand, Michael Wagner

**Affiliations:** ^1^ Brandenburg Medical School (MHB) Neuruppin Germany; ^2^ European University Viadrina Frankfurt (Oder) Germany; ^3^ Berlin School of Economics and Law Berlin Germany; ^4^ Sporthopaedicum Berlin Berlin Germany

**Keywords:** ACL, anteromedial instability, arthroscopy, peroneus longus split graft, SMCL

## Abstract

**Purpose:**

To evaluate 2‐year clinical, radiological, and patient‐reported outcomes after combined anterior cruciate ligament (ACL) reconstruction and single‐strand superficial medial collateral ligament (sMCL) reconstruction using a peroneus longus split graft (PLSG) in patients with anteromedial rotatory instability (AMRI).

**Methods:**

Patients with ACL insufficiency and clinically and intraoperatively confirmed AMRI who underwent combined ACL and sMCL reconstruction using a PLSG were retrospectively identified and prospectively followed. AMRI was diagnosed in this study based on a combination of ACL insufficiency, increased anteromedial drawer in external tibial rotation, medial laxity on valgus stress testing at 20° of knee flexion, pivot‐shift grading as an indicator of overall rotatory instability of the knee, and supportive arthroscopic findings such as the medial meniscus squeeze sign. At a minimum follow‐up of 2 years, 59 of 63 patients (mean age 30 years, 48% female) were available for evaluation. Postoperative stability was assessed using KT‐1000 instrumented side‐to‐side measurements for the Lachman test and anterior drawer test at 90° flexion with neutral and 15° external tibial rotation (AMRI‐related). Pivot‐shift grading and valgus stress radiographs obtained at 20° of knee flexion were additionally performed. Patient‐reported outcome measures (PROMs) included International Knee Documentation Committee (IKDC) Subjective, Lysholm, Tegner activity scale, and return‐to‐any‐sport rate. A non‐inferiority margin of 3 mm for side‐to‐side differences was predefined.

**Results:**

The mean side‐to‐side difference for the AMRI‐related anterior drawer was 0.0 ± 1.3 mm (95% CI: −0.4 to 0.3 mm), meeting the predefined non‐inferiority criterion (upper one‐sided 95% confidence limit 0.3 mm; *p* < 0.001). The mean Lachman side‐to‐side difference was 1.8 mm ± 1.3 mm and remained within clinically acceptable limits. A negative pivot‐shift was observed in 93% of patients. IKDC (59 ± 20 to 85 ± 12; *p* < 0.001) and Lysholm (61 ± 25 to 89 ± 11; *p* < 0.001) scores improved significantly, whereas the Tegner Activity Scale (5.8 ± 1.7 to 5.2 ± 1.6; *p* = 0.007) showed a small but significant decrease. The mean side‐to‐side difference in medial joint opening on valgus stress radiographs was 0.6 mm ± 1.0 mm, remaining below the predefined clinical reference threshold. At 2 years, 66% of patients had returned to any sports.

**Conclusion:**

Combined ACL and single‐strand sMCL reconstruction using a PLSG was associated with low postoperative side‐to‐side differences, satisfactory postoperative stability, favourable postoperative PROMs and low ankle donor‐site morbidity at 2‐year follow‐up. This tunnel‐sparing technique may be considered a reasonable surgical option for patients with ACL insufficiency and clinically relevant concomitant AMRI.

**Level of Evidence:**

Level IV.

**Abbreviations**
ACL: anterior cruciate ligament
ADT: anterior drawer test
AMRI: anteromedial rotatory instability
AOFAS: American Orthopaedic Foot & Ankle Score
CI: confidence interval
dMCL: deep medial collateral ligament
HT: hamstring tendons
IKDC: International Knee Documentation Committee
MCID: minimum clinically important difference
MRI: magnetic resonance imaging
PASS: patient acceptable symptom state
PCL: posterior cruciate ligament
PLSG: peroneus longus split graft
POL: posterior oblique ligament
PROM: patient reported outcome measures
ROM: range of motion
RTS: return to sports
SD: standard deviation
sMCL: superficial medial collateral ligament
ST: semitendinosus tendon

## INTRODUCTION

Injuries to the medial capsuloligamentous complex are frequent concomitant findings in anterior cruciate ligament (ACL) ruptures, with reported coincidence rates up to 70% [41, 51, 55]. These injuries contribute to anteromedial rotatory instability (AMRI), with a clinically relevant pattern of combined valgus laxity and external tibial rotation [29, 41, 53].

The primary restraints include the superficial medial collateral ligament (sMCL)—the principal valgus stabiliser throughout higher degrees of flexion—and the deep medial collateral ligament (dMCL), which primarily limits external tibial rotation at deeper flexion angles [6, 41, 54]. The posterior oblique ligament (POL) contributes mainly posteromedial stability near extension, particularly as a restraint to internal rotation and valgus laxity [36]. More recently, the anterior oblique ligament (AOL) has been described by Jorge et al. as a distinct anteromedial structure, although its relationship to the dMCL remains debated [31].

Clinically, AMRI is characterised by an anteromedially accentuated anterior drawer in external tibial rotation and increased valgus stress laxity, particularly in 20°−30° flexion [29]. In ACL‐deficient knees, pivot‐shift grading may additionally indicate overall rotatory instability, but it should not be interpreted as a specific stand‐alone diagnostic test for AMRI.

From a pathomechanical perspective, AMRI shifts the knee joint centre of rotation laterally [6], potentially accelerating medial compartment degeneration [49, 52]. While many concomitant medial injuries can be treated conservatively, surgical reconstruction is indicated when clinical instability persists, particularly in grade III injuries with combined rotational valgus laxity [37, 41].

Isolated ACL reconstruction may be insufficient to adequately control AMRI [7, 9, 17, 19, 25, 41]. Initial clinical studies have reported encouraging results following combined ACL and medial reconstruction procedures [1, 5, 12, 28]. At the same time, the currently available literature increasingly includes reconstruction concepts specifically targeting the anteromedial structures, including AOL‐reconstructions, combined dMCL and sMCL concepts and other multi‐structure techniques [14, 27, 30, 42, 46, 50].

The purpose of this study was to evaluate clinical stability, functional outcomes, and patient‐reported satisfaction following combined ACL and single‐strand sMCL reconstruction using a peroneus longus split graft (PLSG).

We hypothesised that this technique would result in clinically equivalent stability to the contralateral healthy knee within a predefined non‐inferiority margin of 3 mm side‐to‐side difference.

## METHODS

### Patient selection

Patients with ACL insufficiency and concomitant AMRI treated with the surgical technique described below were retrospectively identified from an institutional database. All procedures were performed between March 2020 and March 2023 at a single institution. Postoperative follow‐up examinations and outcome assessments were performed prospectively to a standardised protocol.

Inclusion criteria comprised patients aged between 18 and 50 years who underwent combined ACL reconstruction and single strand sMCL reconstruction for clinically diagnosed AMRI and consented to participate in the study. Exclusion criteria included concomitant posterior cruciate ligament (PCL) injuries, bucket‐handle or root tears of the menisci of the ipsilateral knee joint, as well as prior injury or previous surgical procedures involving the ACL, PCL, or medial collateral ligament on the contralateral side.

All patients underwent standardised preoperative magnetic resonance imaging of the affected knee. MRI was not used as the sole criterion for surgical indication but served as a supportive tool to document injury of the medial structures and associated intra‐articular pathology. Patients undergoing revision or re‐revision ACL reconstruction were included if they demonstrated clinically relevant AMRI at the time of the index procedure. A standardised distinction between acute and chronic medial insufficiency in these revision cases was not consistently available and was therefore not analysed separately.

### Diagnosis of AMRI

The indication for additional sMCL reconstruction in this study was based on a combination of clinical examination under anaesthesia and intraoperative arthroscopic assessment. Clinical criteria included (1) a positive pivot‐shift test (grade II or higher), (2) increased anterior tibial translation on the anteromedial drawer test at 90° of flexion with 15° of external tibial rotation compared with both the contralateral knee and neutral tibial rotation [56], and (3) valgus laxity at 20° of knee flexion (grade I or higher). The pivot‐shift test was interpreted as an indicator of overall rotatory instability in the ACL‐deficient knee and not as a specific stand‐alone diagnostic test for AMRI. Accordingly, the indication for sMCL reconstruction was not based on the pivot‐shift test alone, but on the combined clinical and intraoperative findings described above.

In the healthy knee, anterior tibial translation during external tibial rotation is typically lower than in neutral rotation; therefore, similar or increased translation in external rotation compared with neutral rotation was considered suggestive of AMRI.

The indication for surgical treatment was further supported intraoperatively by a positive medial meniscus squeeze sign.

### Surgical procedure

All procedures were performed by a single experienced surgeon (MW). After confirmation of the indication, ACL reconstruction was performed using the ipsilateral or contralateral semitendinosus tendon (ST) autograft in primary cases and a soft‐tissue all‐inside quadriceps tendon (QT) autograft in selected revision cases in which the ST was unavailable because of previous harvest. In addition, a PLSG was harvested through a separate minimally invasive incision on the distal lower leg (Figure 1a–c). The PLSG was chosen because it provides adequate graft length and diameter while being associated with low donor‐site morbidity [28, 39, 57, 58].

**Figure 1:**
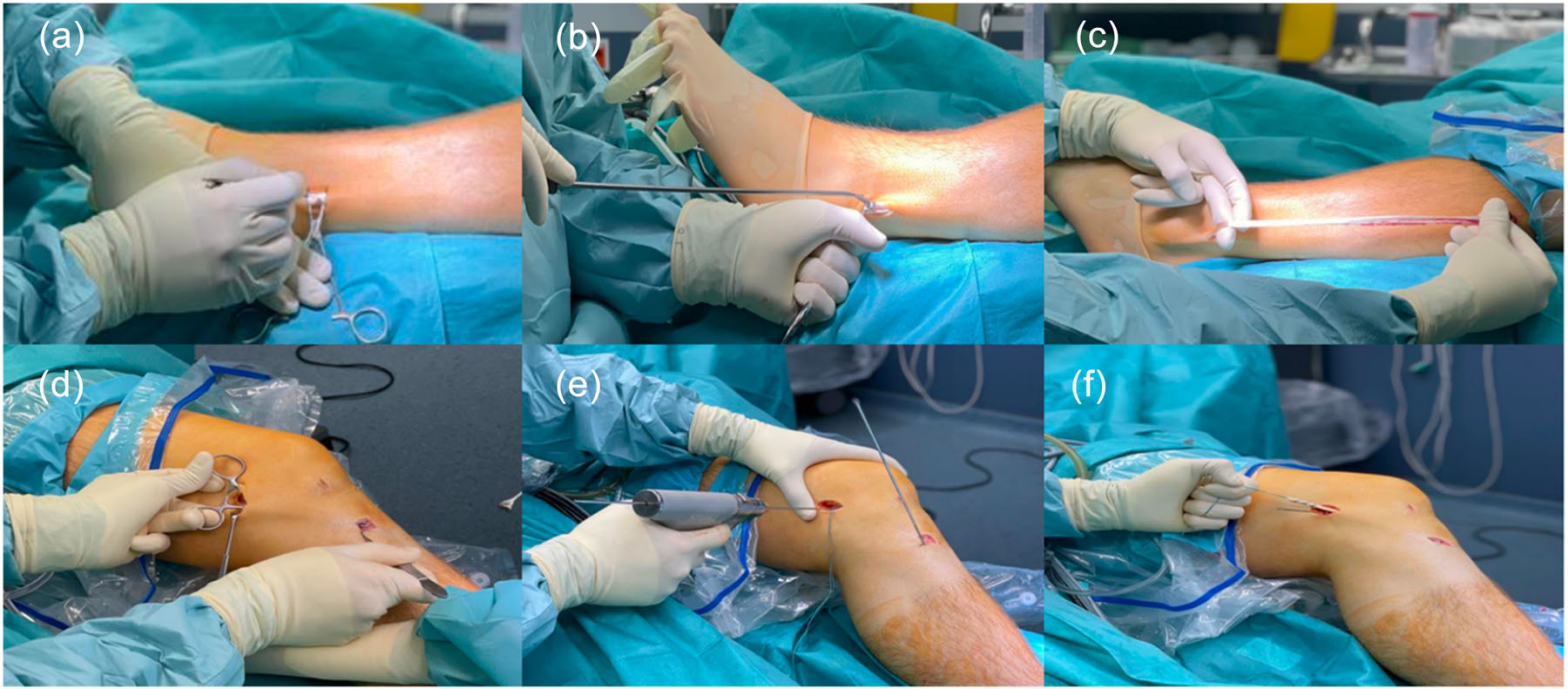
Intraoperative steps of peroneus longus split graft harvesting and superficial medial collateral ligament (sMCL) reconstruction. (a) Minimally invasive incision proximal to the retinaculum and slightly dorsal to the fibula and longitudinal splitting of the peroneus longus tendon. (b) Harvesting of the anterior portion of peroneus longus split graft using a tendon stripper. (c) Harvested peroneus longus split graft. (d) Passage of a shuttle suture beneath the sartorius fascia toward the tibial superficial medial collateral ligament insertion. (e) Assessment of graft isometry using temporary guidewire placement. (f) Final graft fixation following femoral and tibial tunnel preparation.

After completion of ACL reconstruction, anteromedial stabilisation was performed.

A medial approach was used to expose the femoral footprint of the sMCL while carefully preserving the saphenous nerve. A soft‐tissue tunnel was then created beneath the sartorius fascia towards the tibial sMCL insertion (Figure 1d).

The femoral and tibial attachment sites were identified based on anatomic landmarks, as shown in Figure 2, without fluoroscopic guidance. The femoral fixation point was posterior to the medial epicondyle and distal to the adductor tubercle. The tibial fixation point was positioned within the anterior portion of the distal tibial sMCL attachment beneath the sartorius fascia. Temporary guidewires were placed to the presumed insertion sites and graft isometry was assessed throughout the range of motion (Figure 1e) before definitive tunnel creation. A slight tension in full extension with progressive relaxtion during flexion was considered appropriate. Thus, definitive tunnel positioning was based on a combination of anatomic landmarks and intraoperative assessment of graft tension behaviour rather than on predefined radiographic coordinates. The graft was fixed on the tibial side using a suspensory fixation device and on the femoral side using an interference screw with the knee positioned at approximately 20°–30° of flexion and slight internal rotation (Figure 1f). This position was chosen to reduce residual external tibial rotation and anteromedial drawer laxity after reconstruction. Graft tensioning was performed manually until the graft was taut without excessive medial tightening. No standardised tensiometer or defined fixation force was used.

**Figure 2:**
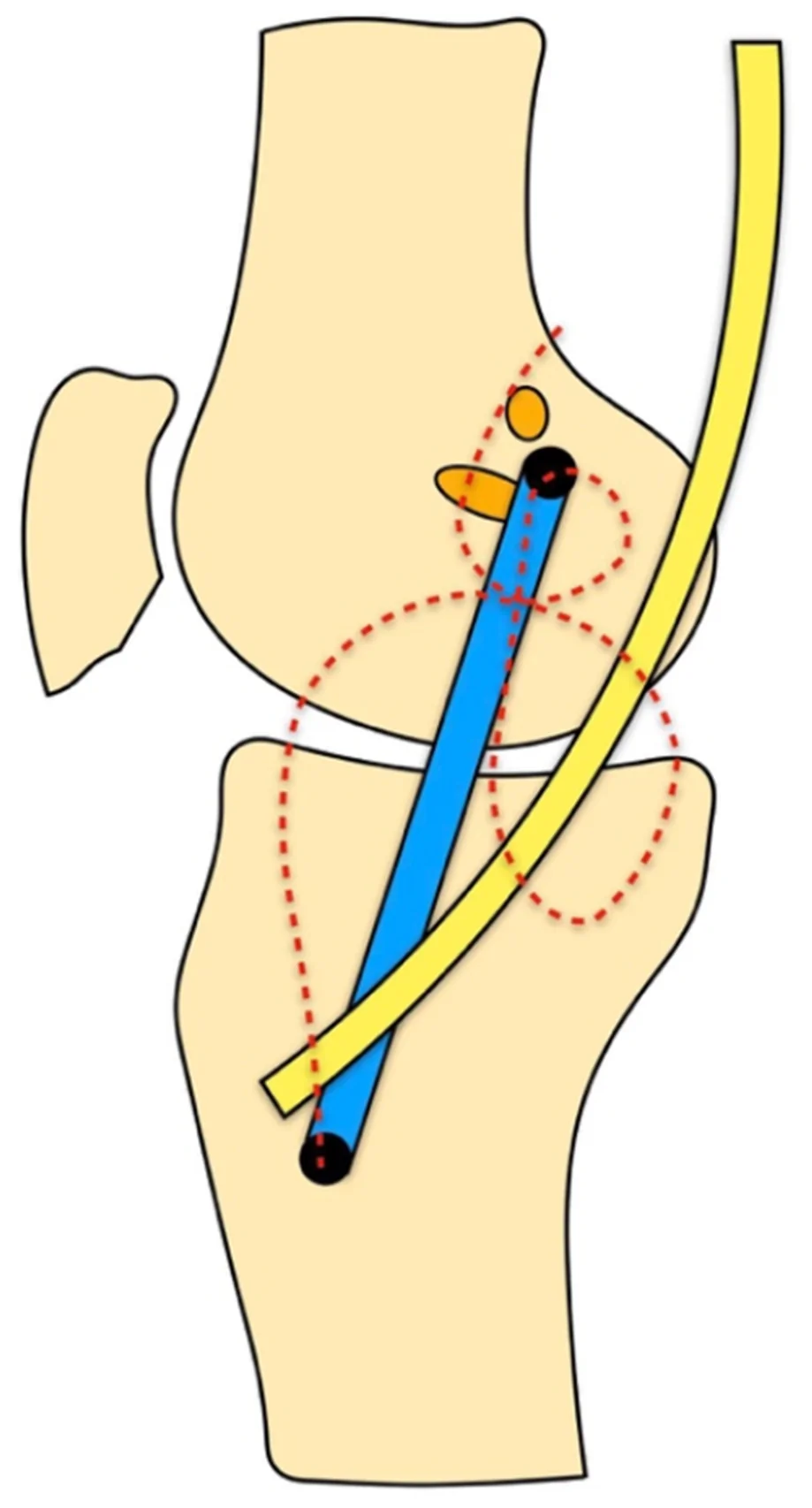
Schematic medial view showing the femoral and tibial tunnel positions for anterior single‐strand superficial medial collateral ligament (sMCL) reconstruction. The blue band represents the reconstructed sMCL graft along its intended course beneath the sartorius fascia. Black circles indicate the femoral and tibial fixation points. The femoral fixation point is positioned posterior to the medial epicondyle and distal to the adductor tubercle. The tibial fixation point is positioned in the anterior portion of the distal tibial sMCL attachment. Orange outlines indicate the medial femoral condyle and the adductor tubercle/medial epicondyle region used as anatomic landmarks for orientation. The red dashed line represents the Burmester curve used as a conceptual guide for near‐isometric graft orientation. The yellow band represents the sartorius muscle.

Additional anterolateral stabilisation was performed only in selected cases, when pronounced anterolateral rotatory laxity was present, as reflected mainly by a high‐grade pivot shift test (≥grade II) and revision ACL reconstruction and patient‐specific risk factors (young age, pivoting sports). A modified Lemaire lateral extraarticular tenodesis was used.

### Postoperative rehabilitation

Postoperative rehabilitation followed the institutional standard protocol and did not include bracing or range‐of‐motion restrictions. Partial weight‐bearing with two forearm crutches was allowed for up to 2 weeks, depending on the patient's recovery and ability to ambulate safely without crutches. Supervised physiotherapy was started immediately after surgery. Progression to sport‐specific activities was individualised and based on clinical and physiotherapist‐guided criteria, including full range of motion, absence of pain and effusion, restoration of clinical stability, normalised gait and movement pattern and progressive neuromuscular control. Low‐risk non‐contact sport‐specific activities were considered after approximately 3–4 months only when these criteria were fulfilled. Return to contact or pivoting sports was delayed and individualised, generally not before 8–9 months. Formal objective muscle strength testing, such as standardised isokinetic testing or predefined limb symmetry index, was not systematically available in this cohort.

In patients with concomitant meniscal repair, postoperative rehabilitation was adapted according to the respective meniscal procedure (e.g., range‐of‐motion and weight‐bearing restrictions).

### Data collection

Preoperative knee stability was assessed under anaesthesia using standardised clinical examination with side‐to‐side comparison. The assessment included evaluation of knee effusion, range of motion (ROM), Lachman test, pivot shift test, valgus laxity at 0° and 20° of knee flexion, anterior drawer test (ADT) at 90° of knee flexion and 15° external and 15° internal tibial rotation, as well as varus instability at 0° and 20° of knee flexion.

Postoperative data collection was performed in a standardised manner after 6, 12, and 24 months using a structured examination protocol. All postoperative clinical follow‐up examinations were performed by a single examiner (PW). Formal intra‐rater reliability was not assessed.

Clinical assessment included the presence of knee effusion, ROM, Lachman test, ADT in 90° flexion, ADT and 15° external tibial rotation, pivot shift test, and evaluation of medial joint opening at 0° and 20° of knee flexion using side‐to‐side comparison.

Preoperative clinical examinations were performed under anaesthesia, whereas all postoperative assessments were conducted with the patient awake.

Instrumented stability measurements (Lachman test, ADT with neutral tibial rotation, and ADT with 15° external tibial rotation) were obtained using a KT‐1000 arthrometer under maximum manual load.

Because no validated instrumented test specifically exists for quantifying AMRI in vivo, anterior tibial translation measured at 90° of knee flexion with 15° of external tibial rotation was used as a pragmatic surrogate of AMRI.

Three measurements were recorded at each time point, and the median value was used for analysis to reduce measurement variability. Side‐to‐side differences were defined as the difference between the operated and contralateral knee and classified according to the IKDC criteria (A: ≤2 mm; B: 3–5 mm; C: ≥6 mm).

Patient‐reported outcome measures (PROMs) included the IKDC Subjective Score, Lysholm Score and Tegner Activity Scale. Preoperative PROMs were obtained retrospectively at the 2‐year follow‐up using a standardised questionnaire. Because this approach is susceptible to recall bias, pre‐to‐postoperative PROM comparisons were considered exploratory and interpreted cautiously. 2‐year PROMs were available for the full cohort and served as the primary PROM assessment. 1‐year PROM data were available only for a subset of patients; these data were therefore treated as exploratory and are reported descriptively only. For responder analysis, previously published threshold values for clinically relevant change were applied (11.5 points for the IKDC Score and 8.9 points for the Lysholm Score) [16, 44].

In addition, the American Orthopedic Foot & Ankle Score (AOFAS) was collected to evaluate ankle‐related outcomes following PLSG harvesting.

At the 2‐year follow‐up, return to any sports, donor‐site‐related complaints, and wound healing at the knee and ankle joint were documented.

Radiological evaluation was performed at 2‐year follow‐up using bilateral valgus stress radiographs at 20° of knee flexion with a standardised Telos device (15 kPa). Medial joint space width was measured digitally on both sides by the same examiner.

Because preoperative stability was assessed clinically under anaesthesia, while postoperative follow‐up was performed in awake patients using standardised instrumented measurements, direct statistical comparison between pre‐ and postoperative stability was not feasible. They were analysed descriptively to characterise baseline findings and postoperative stability of the cohort. Statistical comparisons were performed exclusively between the operated knee and the healthy contralateral side to evaluate postoperative stability outcomes.

The study protocol was approved by the institutional ethics committee of the Brandenburg Medical School (approval number: E‐02‐20221124, 19 December 2022).

### Statistics

The data were recorded pseudonymously in Excel (version 16.98) and analysed using R (R 4.5.1 GUI 1.82).

The primary outcome was the side‐to‐side difference in AMRI‐related laxity, assessed by anterior tibial translation at 90° of knee flexion with 15° of external tibial rotation. AMRI was considered a combined instability pattern involving anterior translation, external tibial rotation and valgus laxity rather than isolated medial opening. As no validated quantitative threshold for AMRI currently exists, a non‐inferiority margin of 3 mm was predefined as a conservative reference for clinically acceptable side‐to‐side laxity, informed by established thresholds for anterior translation and medial instability [20, 23, 36], but not intended as a validated AMRI cut‐off.

Postoperative stability outcomes were evaluated by comparing the operated knee with the healthy contralateral knee. Normality of continuous variables was assessed using the Shapiro–Wilk test. Depending on data distribution, Wilcoxon signed‐rank tests were used for side‐to‐side comparisons. Paired two‐tailed *t*‐tests were additionally performed as a robustness analysis. Subgroup analyses (primary ACL rupture, ACL revision, ACL‐re‐revision) were conducted using the Wilcoxon signed‐rank tests and postoperative differences between subgroups were assessed using the Kruskal–Wallis test.

Non‐inferiority analysis was assessed by calculating the one‐sided upper 95% confidence limit of the mean side‐to‐side difference and by performing a one‐sided *t*‐test against the predefined non‐inferiority margin of 3 mm, reflecting equivalence to the healthy contralateral knee within a clinically acceptable range rather than comparison to an external control group. To account for the moderate sample size and the non‐normally distributed differences, 95% confidence intervals (CIs) were additionally estimated using a non‐parametric bootstrap procedure with 5000 resamples for further robustness testing.

Pre‐ and post‐operative PROMs were compared using Wilcoxon signed‐rank tests. Postoperative group comparisons were performed using the Kruskal–Wallis test. Statistical significance was set at *p* < 0.05.

At the time of study planning, no reliable estimates of variability were available from the literature for the primary outcome (AMRI). Therefore, no a priori sample size calculation could be performed for this outcome. After completion of data collection, the observed variability of the primary outcome was evaluated descriptively to assess whether the achieved sample size was appropriate. The observed standard deviation of the side‐to‐side difference was 1.6 mm, indicating that the available cohort size provided sufficient precision for the estimation of postoperative stability outcomes. Accordingly, the final sample of 59 patients with complete follow‐up was considered methodologically adequate.

## RESULTS

*Study cohort and follow‐up:* A total of 63 patients were included in the study. The mean age was 30.0 ± 7.2 years, and 30 patients (48%) were women and 33 (52%) were men.

Primary ACL rupture was present in 30 patients (47.6%), ACL revision in 26 patients (41.3%), and ACL re‐revision in 7 patients (11.1%). Given the heterogeneity of the cohort, particularly with regard to primary, revision and re‐revision ACL reconstruction, subgroup analyses were considered exploratory and are reported with caution. In patients undergoing revision or re‐revision ACL reconstruction, AMRI was diagnosed clinically at the time of the index procedure; however, a separate classification into acute and chronic medial insufficiency was not consistently available.

For ACL reconstruction, the ipsilateral ST was used in 32 cases (51%), the contralateral ST in 25 cases (40%) and the QT in 6 cases (9%).

Meniscal pathology was documented in 26 patients (41.3%), predominantly involving the medial meniscus. Single strand sMCL reconstruction using PLSG was performed in all cases. Additional anterolateral stabilisation using a modified Lemaire lateral extraarticular tenodesis was performed in 18 of 63 cases (28.6%).

Sixty‐three patients were enrolled in the study. The follow‐up rate among enrolled patients was 94% (59/63). Two patients (3%) were lost to follow‐up, and two patients (3%) were excluded because of ACL re‐rupture and contralateral ACL rupture. Five reoperations were performed during follow‐up (8.5%), including two cyclops resections, two wound revisions at the PLSG harvest site, and one arthrolysis for arthrofibrosis. The final analysis therefore comprised 59 patients with a mean follow‐up of 26.4 ± 3.5 months. Patient flow is illustrated in Figure 3.

**Figure 3:**
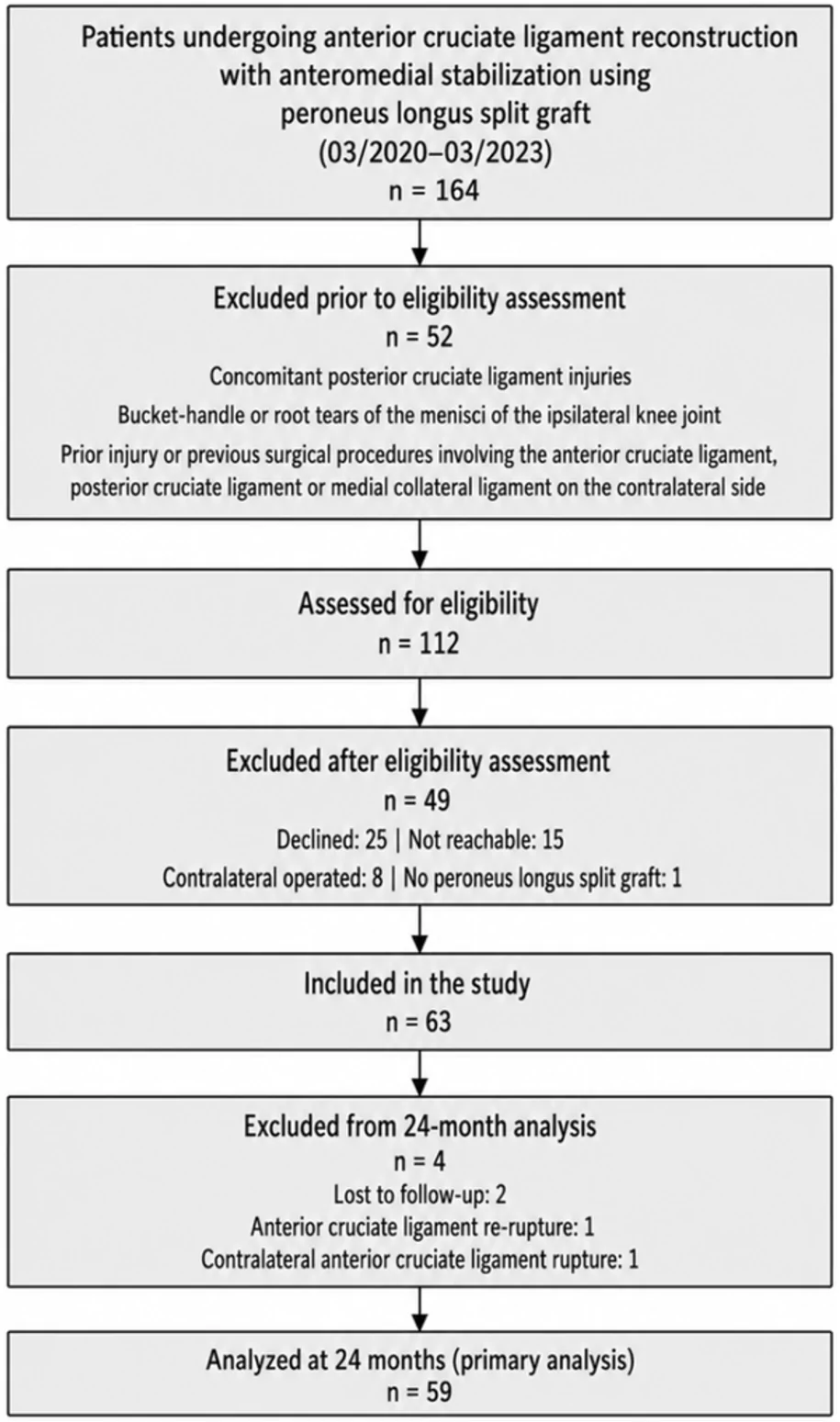
Flow diagram illustrating patient inclusion, exclusion and follow‐up.

### Clinical stability

Because preoperative stability was assessed under anaesthesia without instrumented measurements and postoperative stability was assessed in awake patients using standardised clinical and instrumented measurements, direct statistical comparison between preoperative and postoperative stability was not performed. Postoperative stability was evaluated by side‐to‐side comparison with the contralateral healthy knee.

For the primary outcome, the mean side‐to‐side difference for the AMRI‐related ADT at 90° of knee flexion with 15° of external tibial rotation was 0.0 ± 1.3 mm (95% CI, −0.4 to 0.3 mm; *p* = 0.743). The predefined non‐inferiority criterion of 3 mm was met, with an upper one‐sided 95% confidence limit of 0.3 mm (*p* < 0.001). Bootstrap analysis confirmed these findings (95% CI, −0.4 to 0.3 mm). No significant between‐group differences were observed in exploratory subgroup analyses.

For secondary stability outcomes, the mean side‐to‐side difference in the Lachman test was 1.8 ± 1.3 mm (95% CI, 1.4–2.1 mm; *p* < 0.001), favouring the contralateral knee, but remained below the predefined non‐inferiority threshold. The ADT in neutral tibial rotation showed a mean side‐to‐side difference of 0.2 ± 1.1 mm (95% CI, −0.1 to 0.5 mm; *p* = 0.12) and likewise met the non‐inferiority criterion. Detailed postoperative stability data are provided in Table 1.

**Table 1:** Postoperative stability outcomes at 2‐year follow‐up.

Outcome	Operated knee, mean ± SD	Contra‐lateral knee, mean ± SD	Side‐to‐side difference, mean ± SD	*p*‐value	Between‐group *p*‐value^a^	Non‐inferiority *p*‐value^b^	Upper one sided 95% CL
AMRI‐related anterior drawer test (external tibial rotation), mm	3.8 ± 1.3	3.8 ± 1.3	0.0 ± 1.3	N.S.	N.S.	<0.001	0.25
Anterior drawer test (neutral tibial rotation), mm	4.5 ± 1.3	4.2 ± 1.3	0.2 ± 1.1	N.S.	N.S.	<0.001	0.48
Lachman test, mm	9.9 ± 2.3	8.1 ± 2.4	1.8 ± 1.3	<0.001	N.S.	<0.001	2.05

*Note*: Values are presented as mean ± SD otherwise stated. Side‐to‐side differences were calculated as operated minus contralateral knee.

Abbreviations: ACL, anterior cruciate ligament; CL, confidence limit; N.S., non‐significant; SD, standard deviation.

^a^ Between‐group *p*‐values refer to exploratory subgroup comparisons among primary ACL rupture, ACL revision and ACL re‐revision cases (Kruskal–Wallis test).

^b^ Non‐inferiority was tested against the predefined threshold of 3 mm (one‐sided *t*‐test).

Preoperatively, most patients demonstrated grade II or grade III pivot‐shift instability. At 2‐year follow‐up, 93.2% of patients had a negative pivot‐shift test and 6.8% had grade I instability (*p* < 0.001). Medial valgus instability was present preoperatively in 74.6% of knees in full extension and in 93.7% at 20° of knee flexion. Postoperatively, all patients were stable in full extension and 98.3% were stable under valgus stress at 20° of knee flexion.

Radiological evaluation: At 2‐year follow‐up, valgus stress radiographs at 20° of knee flexion demonstrated a mean side‐to‐side difference in medial joint space opening of 0.6 ± 1.0 mm (95% CI, 0.4–0.9 mm), favouring the contralateral knee. Although this difference was statistically significant (*p* < 0.001), it remained below the predefined non‐inferiority threshold of 3 mm, as illustrated in Figure 4. No postoperative differences in medial joint space opening were observed between subgroups.

**Figure 4:**
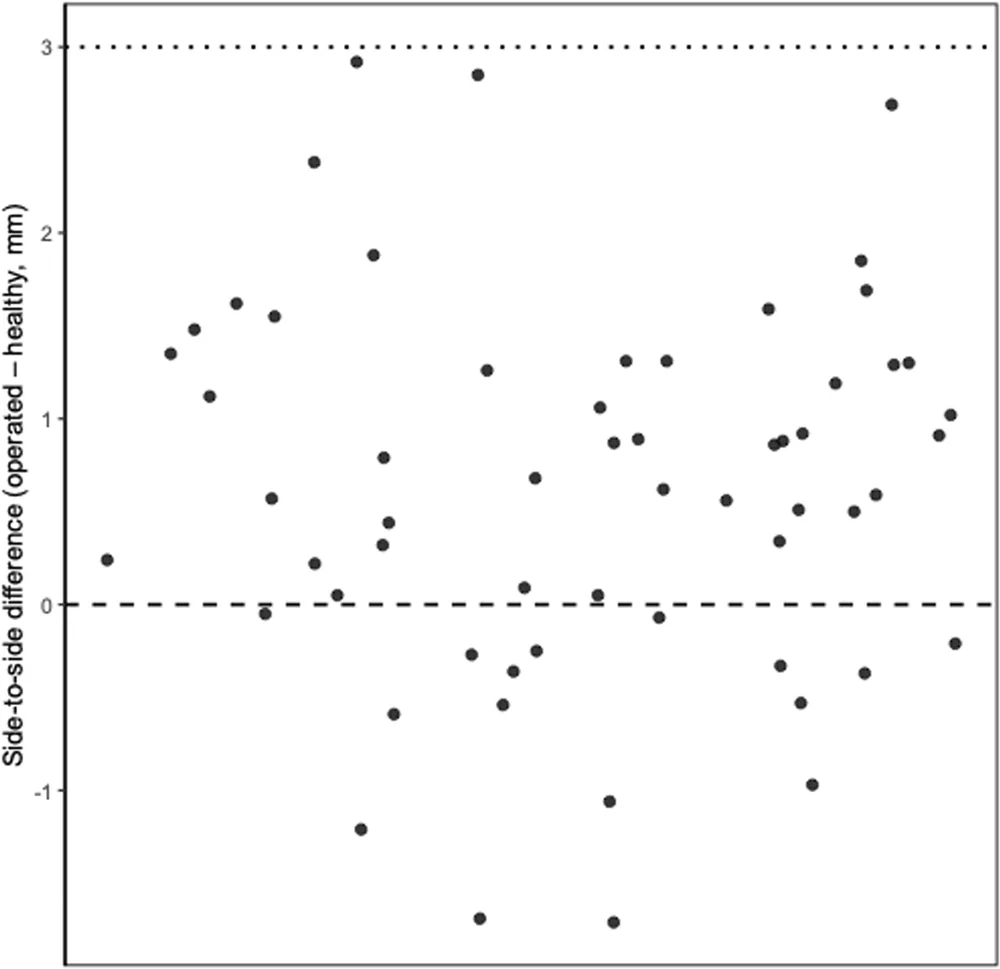
Valgus stress radiographs at 20° of knee flexion. Side‐to‐side differences in medial joint space opening (operated—contralateral knee; mm) are shown. The *x*‐axis represents individual patients sorted by side‐to‐side difference. The dashed horizontal reference line indicates 0 mm; and the dotted line indicates the predefined non‐inferiority margin of 3 mm.

PROMs: The IKDC Subjective Score improved from 58.5 ± 20.4 preoperatively to 85.4 ± 11.6 at follow‐up (*p* < 0.001). The Lysholm Score increased from 60.9 ± 24.5 to 88.5 ± 11.0 points (*p* < 0.001). The Tegner Activity Scale decreased from 5.8 ± 1.7 to 5.2 ± 1.6 points (*p* = 0.007). No significant postoperative differences in PROMs were observed between subgroups. Detailed PROM data are summarised in Table 2, with exploratory 1‐year PROM data reported for a subset of 40 patients only.

**Table 2:** Supplementary patient‐reported outcome details.

Outcome	Change 1 year (*n* = 40; mean ± SD)	Change 2 years (*n* =59; mean ± SD)	Overall *p*‐value^a^	Effect size^b^	Responder rate^c^	Between‐group *p* value^d^
International Knee Documentation Committee (IKDC) subjective score	+14.5 ± 20.0	+26.9 ± 21.4	<0.001	1.26	79.7% ≥ 11.5‐point threshold	N.S.
Lysholm score	+27.5 ± 24.5	+28.0 ± 22.0	<0.001	1.09	76.3% ≥ 8.9‐point threshold	N.S.
Tegner activity scale	Preoperative median 6 (interquartile range [IQR], 4–7); median 4 (IQR, 4–7) at 1 year	Preoperative median 6 (IQR, 4–7); median 5 (IQR, 4–7) at 2 years	0.007	−0.36	Improved by ≥ 1 point: 13.6%; unchanged: 45.8%; reduced by ≥1 point: 40.7%	N.S.

^a^ Overall *p*‐values refer to preoperative‐to‐2‐year postoperative comparisons and were calculated using the Wilcoxon signed‐rank test. One‐year data are reported descriptively only.

^b^ Effect sizes are reported as Cohen's *d* for IKDC and Lysholm and as Cohen's *dz* for the Tegner activity scale.

^c^ Responder rates refer to the 2‐year follow‐up. Predefined thresholds for clinically relevant change were 11.5 points for IKDC and 8.9 points for Lysholm.

^d^ Between‐group *p*‐values refer to exploratory subgroup comparisons among primary anterior cruciate ligament (ACL) rupture, ACL revision and ACL re‐revision cases at 2‐year follow‐up.

Abbreviation: N.S. = non‐significant.

*Return to any sport:* At 2‐year follow‐up, 39 of 59 patients (66.1%) had returned to any sport. Return to any sport was achieved by 59% of patients with primary ACL rupture, 70% with ACL revision, and 86% of those undergoing ACL re‐revision; however, these subgroup data should be interpreted with caution because of the small sample size and the heterogeneous revision setting.

*Ankle‐related outcomes:* Ankle donor‐site morbidity after PLSG harvesting was low. One patient reported persistent sensory disturbance. Two patients required wound revision because of delayed wound healing; in both cases, the symptoms resolved without long‐term sequelae. Postoperative AOFAS demonstrated good ankle function, with a median score of 100 points (mean ± SD: 96.7 ± 5.1; range 83–100) and 88% of patients achieved scores ≥90 points.

## DISCUSSION

The main finding of the present study is that combined ACL reconstruction and single‐strand sMCL reconstruction using a PLSG was associated with low postoperative side‐to‐side differences in AMRI‐related laxity, favourable PROMs and low ankle donor‐site morbidity at 2‐year follow‐up. Given the absence of preoperative instrumented stability measurements and the lack of a control group, these findings should be interpreted as evidence of satisfactory postoperative stability and clinical feasibility rather than proof of superiority or direct pre‐to‐post improvement.

AMRI is a complex instability pattern that combines anterior translation of the medial tibial plateau, external tibial rotation and valgus laxity [29, 41]. Current biomechanical studies have demonstrated that both the sMCL and dMCL contribute to the control of AMRI. The sMCL acts as the principal valgus stabiliser and an important restraint to anteromedial laxity, whereas the dMCL contributes particularly to the control of external tibial rotation [6, 8, 41, 54]. More recently, the AOL has been described as a distinct anteromedial structure, although its anatomical and functional relationship to the dMCL remains unresolved [31].

Therefore, the present study should not be interpreted as an anatomic structure‐specific reconstruction model of the sMCL, but rather as a pragmatic clinical stabilisation strategy for ACL‐deficient knees with clinically relevant combined AMRI. This distinction is important when comparing the present technique with currently described reconstruction concepts. Recent techniques increasingly aim to reconstruct the anteromedial complex more anatomically, including AOL‐based procedures, dMCL‐oriented reconstructions or combined multi‐structure concepts [24, 27, 28, 30, 32, 42, 46, 50]. Such approaches may provide biomechanical advantages, especially for rotational control, but they usually require additional tunnels and greater technical complexity, with a potential risk of tunnel convergence in combined ligament surgery [22, 43]. In contrast, the present technique is intentionally tunnel‐sparing and surgically less complex. The current findings suggest that this simplified construct may still provide clinically acceptable postoperative stability, even if it does not anatomically reproduce every component of the anteromedial complex.

A key question is why an isolated sMCL reconstruction could restore stability if the dMCL is considered highly relevant for AMRI control. In our view, the most plausible explanation is that clinical AMRI in ACL‐deficient knees rarely reflects isolated insufficiency of a single medial structure. Rather, it more likely represents a combined functional failure of the medial stabilising complex. Reconstruction of the sMCL may therefore restore a substantial proportion of stability by restoring valgus restraint, reducing secondary anterior translation of the medial tibial plateau and allowing the reconstructed ACL and the remaining structures to function under more favourable loading conditions. In vivo, this effect is likely augmented by contributions from the capsule, meniscocapsular structures, POL and neuromuscular control [33, 48]. Accordingly, our results should not be interpreted as evidence that the dMCL is unimportant, but rather that a clinically simple sMCL‐based construct may be sufficient to achieve acceptable stability.

The present findings also need to be interpreted considering our treatment strategy for AMRI. Although some patients demonstrated valgus laxity in full extension preoperatively, this was interpreted as part of combined medial injury pattern and not as an isolated indication for POL reconstruction. In our cohort, POL reconstruction was therefore not routinely performed based on full‐extension valgus laxity alone, but would have required distinct clinical evidence of relevant posteromedial instability beyond the AMRI pattern addressed by combined ACL and single‐strand sMCL reconstruction [18]. Importantly, the POL was not considered a primary restraint to external tibial rotation.

Radiological valgus stress testing demonstrated small side‐to‐side differences that remained below the predefined non‐inferiority threshold. This 3 mm threshold should be interpreted as a conservative reference rather than a validated AMRI‐specific cut‐off, as no accepted in vivo threshold for AMRI currently exists. Nevertheless, the observed values were within the range reported for medial stability after reconstruction and support the impression that the medial side was restored compared to the contralateral healthy knee [15, 23, 35].

With respect to clinical outcome, the PROM improvements and the low donor‐site morbidity support the technique. However, preoperative PROMs were collected retrospectively and are therefore prone to recall bias, so the magnitude of pre‐to‐postoperative change should be interpreted cautiously [21, 38]. Although the preoperative PROMs were lower than the postoperative scores. Consequently, the present data support favourable postoperative PROMs at 2 years, but they do not provide the same level of evidence as prospectively collected baseline‐to‐follow‐up comparisons. Claims of definitive clinical improvement should therefore be avoided.

In addition, 1‐year PROM data were available only for a subset of patients and were therefore reported as exploratory data only. Return‐to‐any‐sport rates were within the range reported after ACL reconstruction [2, 3, 4]. The apparently higher return‐to‐any‐sport rates in revision and re‐revision cases should not be overinterpreted and most likely reflect small subgroup sizes, selection effects and differences in expectations and activity profiles rather than a true advantage of revision settings.

A potential technical concern of the present single‐strand sMCL construct is the risk of over‐constraint, particularly because the graft was fixed in slight internal tibial rotation and tensioned manually. Excessive graft tension of fixation in excessive internal rotation could theoretically restrict physiological external tibial rotation, alter medial compartment loading or produce nonphysiological medial restraint [47]. In the present cohort, no clinically relevant motion restriction suggesting over‐constraint was observed during follow‐up; however, detailed quantitative rotational kinematics were not assessed.

The use of the ST as an ACL graft in cases of concomitant AMRI is controversial. The medial hamstrings (HT) limit ACL loading in terms of anterior translation and tibial rotation [26, 48]. Although their removal can increase AMRI, especially in cases of concomitant lesions of medial structures [34], ACL reconstruction compensates for a large part of the translational and rotational stability, and additive augmentation procedures (e.g., anterolateral tenodesis, over‐the‐top techniques) further improve rotational control [13, 40, 45]. The clinical relevance of HT removal therefore appears to be limited in the setting of modern reconstructions. At the same time, the PLSG represents an attractive graft option for the medial side because of reliable graft dimensions, and it is associated with low donor‐site morbidity [10, 11, 57]. The low rate of harvest‐related complaints and favourable AOFAS values in the present cohort are consistent with this rationale. Nevertheless, the potential influence of ACL graft choice and the additional use of anterolateral tenodesis cannot be excluded and should be considered when interpreting results.

Overall, the present study suggests that single‐strand sMCL reconstruction using a PLSG may represent a pragmatic option for patients with ACL insufficiency and clinically relevant AMRI, particularly when a technically straightforward medial stabilisation strategy is desired. At the same time, the results should be interpreted in the context of the ongoing anatomical and biomechanical debate regarding the relative roles of the sMCL, dMCL, AOL and POL. Future comparative studies will be needed to define which patients can be treated sufficiently with a simplified sMCL‐based reconstruction and which may benefit from more anatomy‐specific anteromedial reconstruction concepts.

### Limitation

This study has several limitations. The cohort was retrospectively identified, the sample size was moderate, and follow‐up was limited to the medium term with no long‐term data on degenerative changes or late instability. No control group or direct comparison with alternative AMRI reconstruction techniques was available. The indication for additional medial stabilisation and for additional anterolateral tenodesis was made individually by the treating surgeon, so selection bias cannot be excluded. Furthermore, some patients underwent additional anterolateral tenodesis and different ACL grafts were used, which may have influenced stability and PROMs; however, meaningful statistical comparisons were limited by the small subgroup sizes. Preoperative instrumented stability measurements and instrumental assessment of ADT with internal tibial rotation were not available. Formal intra‐rater reliability was not assessed. Preoperative PROMs were collected retrospectively and are therefore subject to recall bias. Graft diameter data for ACL and PLSG were not consistently available in the retrospective data set. The subgroup analyses comparing primary ACL reconstruction, ACL revision and ACL re‐revision cases were exploratory and underpowered, small and clinically heterogeneous. Therefore, the absence of statistically significant between‐group differences should not be interpreted as evidence of clinical equivalence. A type II error due to insufficient statistical power is possible.

This was a single‐centre study, which limits generalisability. In addition, revision cases and re‐revision cases were not consistently subclassified into acute versus chronic medial insufficiency. The mean time to return to any sport was not assessed. Finally, the predefined non‐inferiority threshold of 3 mm should be interpreted as a conservative reference rather than a validated AMRI‐specific cut‐off.

## CONCLUSION

Combined ACL and anterior single‐strand sMCL reconstruction using a PLSG was associated with low postoperative side‐to‐side differences, satisfactory postoperative stability, favourable postoperative PROMs and low ankle donor‐site morbidity at 2‐year follow‐up. This tunnel‐sparing technique may be considered a reasonable surgical option for patients with ACL insufficiency and clinically relevant concomitant AMRI.

## AUTHOR CONTRIBUTIONS

**Philipp Weber**: Conceptualisation; data collection; data analysis; manuscript drafting. **Simone Maxand**: Statistical analysis; statistical supervision; manuscript editing. **Michael Wagner**: Conceptualisation; surgical procedures; supervision; critical revision of the manuscript. All authors read and approved the final manuscript.

## FUNDING INFORMATION

The authors have no funding to report.

## CONFLICT OF INTEREST STATEMENT

The authors declare no conflicts of interest.

## ETHICS STATEMENT

Ethic committee of the Brandenburg Medical School (approval number: E‐02‐20221124, 19 December 2022). Written informed consent was obtained from all individual participants included in the study.

## Data Availability

Data are available on request from the authors.
